# Training and learning ultrasound: survey on a sample of Italian students, impact and role inside the core curriculum of degree courses in medicine and surgery

**DOI:** 10.1007/s40477-023-00830-3

**Published:** 2023-10-05

**Authors:** Andrea Boccatonda, Antonio Ursitti, Alessio Frisone, Patrizio D’Alessandro, Maria Teresa Guagnano, Giovanni Iannetti, Cosima Schiavone

**Affiliations:** 1 Internal Medicine, Bentivoglio Hospital, AUSL Bologna, 40010 Bentivoglio, Italy; 2 Internistic Ultrasound Unit, SS Annunziata Hospital, “G. D’Annunzio” University, 66100 Chieti, Italy; 3 Department of Innovative Technologies in Medicine and Dentistry, University of Chieti-Pescara, Via Dei Vestini 31, 66100 Chieti, Italy; 4 Department of Medicine and Aging Science, “Clinica Medica” Institute, ‘SS Annunziata’ Hospital, “G. D’Annunzio” University, 66100 Chieti, Italy; 5 Ospedale S. Spirito, Università Degli Studi Chieti-Pescara, Chieti, Italy

**Keywords:** Ultrasound, Learning, Medicine, Training

## Abstract

**Purpose:**

Ultrasound is essential in the clinical practice of many medical specialties due to non-invasiveness, rapidity of examination, low costs and simplicity. Many specialized companies and universities pointed out its potential as a teaching tool for medical students. The aim of our study is to evaluate the impact of an ultrasound course on a sample of students attending the fourth, fifth and sixth year of the degree course in Medicine, highlighting changing in satisfaction and preparation. Another target is to verify the capability of a course on ultrasound to positively impact on participants knowledge and competences.

**Methods:**

Students attending 6 training courses of Medicine held between 2017 and 2019 were recruited. Five trainings held during an Italian society of ultrasound in medicine and biology (SIUMB) congress, in a session dedicated to students, and one during an elective didactic activity (ADE) held in Chieti University. A questionnaire was given to the students before and after the course, in order to assess the impact of the course on the motivation and knowledge. Moreover, a test was also administered at the end of the theoretical part, with questions relating to the notions learned.

**Results:**

There was an 81% of correct response to the learning questionnaire by calculating the mean of 5 SIUMB courses performed. The students are strongly motivated to continue learning ultrasound already from the beginning of the course, and this result remains unchanged in the questionnaire administered at the end. The interest of students towards this method is high, and they would ultrasound courses within the Medicine degree, even before participating in the training. It was evident how students positively assessed the course in relation to the acquisition of skills and knowledge, albeit with a tendency to acquire more knowledge rather than skills.

**Conclusions:**

Our data support the usefulness of including ultrasound into the curriculum of medical students and on its use as a teaching tool. Students are highly motivated and perceive a significant improvement in both skills and knowledge following the proposed courses. Hands-on part is necessary in the training course on ultrasonography.

## Introduction

In last years, ultrasound undergone significant changes, thus becoming an increasingly versatile diagnostic tool to employ in several settings [1, 2]. Nowadays, it is essential in the clinical practice of many medical specialties due to non-invasiveness, rapidity of examination, low costs and relative simplicity. Some authors defined it as an “echoscope” as a bedside tool, thus replacing or being complementary to a “stethoscope” [3–5].

Many specialized companies and universities pointed out its potential as a tool for medical students to learn both basic subjects, such as anatomy, physiology and pathology, and more advanced ones, such as traumatology, gastroenterology, internal medicine and diagnostics. Ultrasound allows to perform a real-time view of the structures inside human body, not obtainable with other conventional learning methods. Furthermore, the growing technological development made the ultrasound equipment more accessible both economically and dimensionally, up to portable ultrasound scanners that can be used at the patient's bed and therefore extremely easy to handle not only in the diagnostic but also in the teaching field [6, 7].

Therefore in Europe and in the world numerous experimental studies aimed to demonstrate the effectiveness of ultrasound in the educational field and to identify the timing and the methods in which it can be actively included in the study courses [8, 9]. The encouraging results bring this research forward, with the target of standardizing its use in Medicine and Surgery courses.

The aim of our study is to evaluate the impact of an ultrasound course on a sample of students attending the fourth, fifth and sixth year of the degree course in Medicine and Surgery, highlighting changing in satisfaction and preparation. Another target is to verify the capability of a course on ultrasound to positively impact on participants knowledge and competences. In addition, we propose to evaluate differences among participants of different years of course, in order to suggest an ideal moment to include future ultrasound teaching.

## Materials and methods

Students attending 6 training courses of Medicine held between 2017 and 2019 were recruited. Five trainings held during an Italian society of ultrasound in medicine and biology (SIUMB) congress, in a session dedicated to students, and one during an Elective Didactic Activity (ADE) held in Chieti by Professor Cosima Schiavone. SISM (Italian Secretariat for Students in Medicine) was involved in recruiting students, thus allowing to spread the news of the courses in several locations.

The events held during SIUMB congresses were divided into two moments. A first theoretical part carried out through a frontal lesson, in which the students learned basic ultrasonography and its applications to the physiopathology of some organs. A subsequent practical part consisting in a hands-on activity performed in groups of 8–10 people on a voluntary subject. A SIUMB certified tutor (a specialist in ultrasound or an internal medicine physician experienced in ultrasound) explained the principles of the abdominal ultrasound to the students and then guided them in its execution. Finally, the tutor showed some pathological images on dummies that allowed an ultrasound simulation and answered students’ questions. For the latter phase, the students were divided into larger groups, around 15–20 people, given the limited availability of simulators.

Regarding the integrated teaching activity (ADE), similar methods were used on a population of 100 students of the IV and, V and VI years, consisting in a frontal lesson of about 2 h, followed by hands-on training on healthy volunteer subjects. At first, a tutor experienced in ultrasound and certified by SIUMB showed how to perform an abdominal ultrasound, including basic notions of ultrasound anatomy. Subsequently, the students were able to practice in turns on the same subjects. The main differences compared to SIUMB courses were the absence of a simulator and the different trainer/student ratio since that only one ultrasound machine was available.

**Fig. 1:**
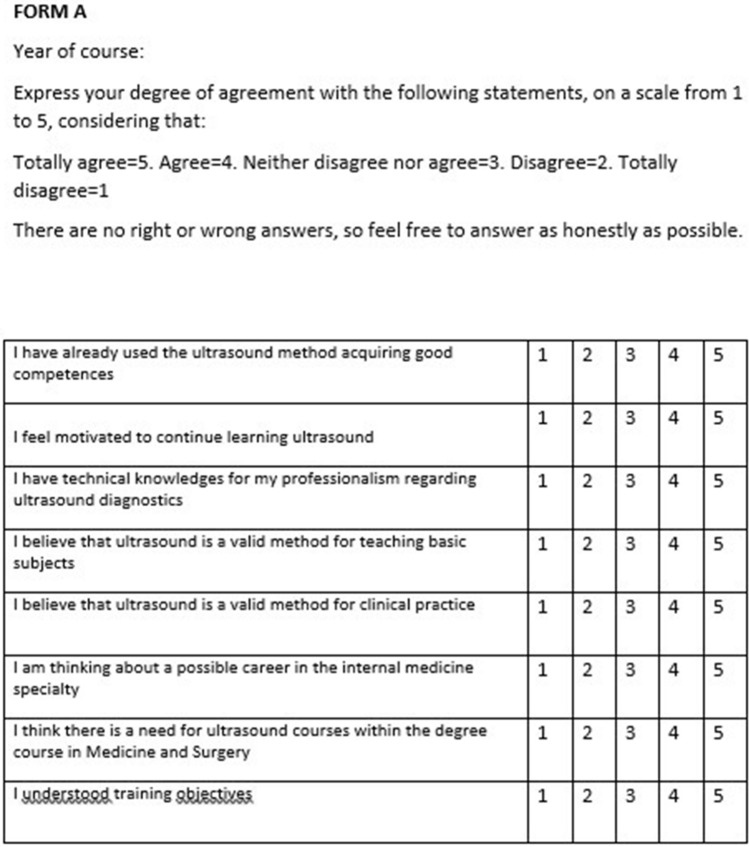
Questionnaire administered before the execution of the Course (FORM A)

**Fig. 2:**
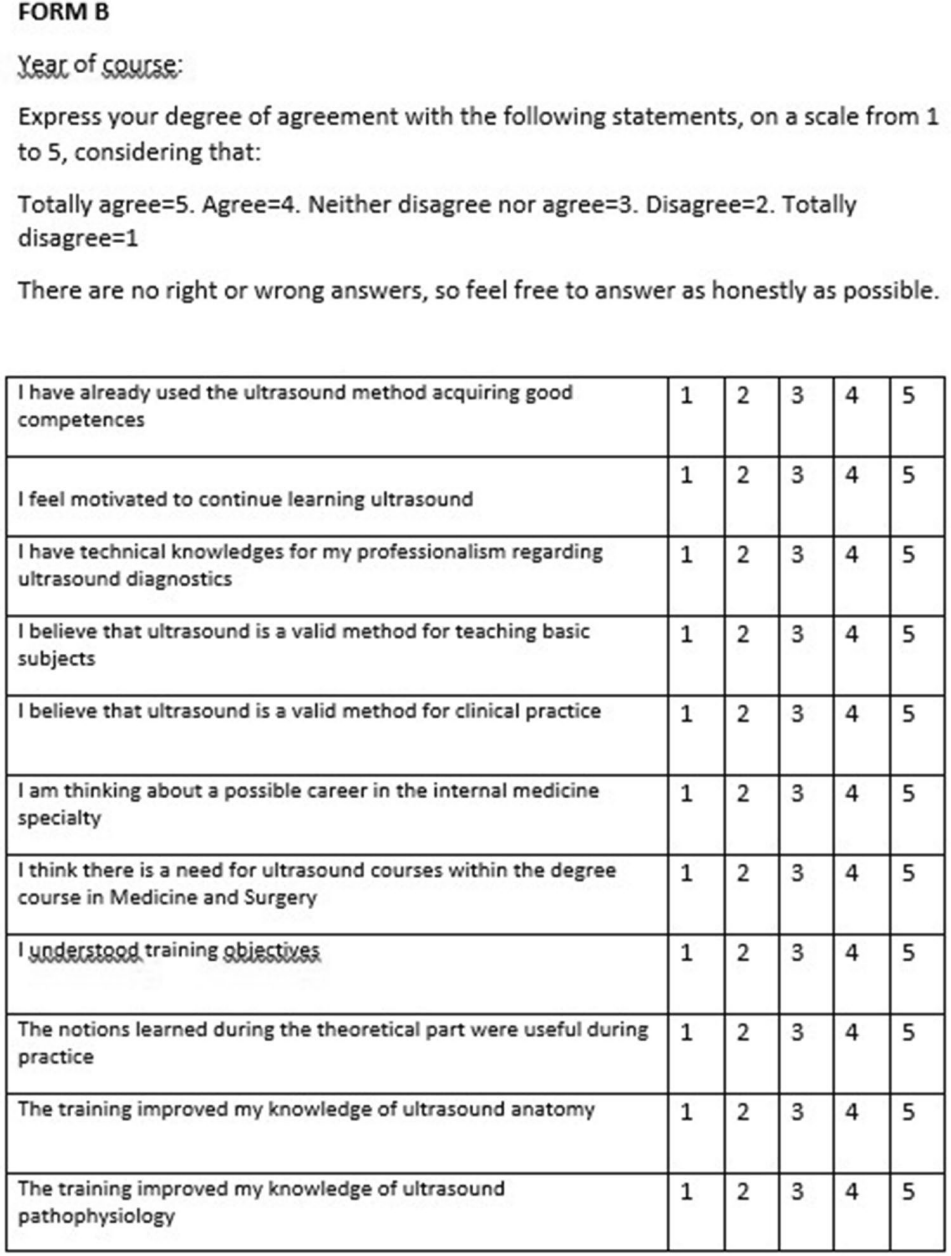
Questionnaire administered after the execution of the Course (FORM B)

A questionnaire was given to the students before the beginning (Form A) and at the end (Form B) of the course, in order to assess the impact of the course on the motivation and knowledge. For this second purpose, a test was also administered at the end of the theoretical part, with questions relating to the notions learned. The questions were proposed by the teachers responsible for the theoretical lessons and reflected the different modules in which they were divided. Form A and B were created on the basis of those already used in other EFSUMB courses, asking the students to indicate the year of the course and to give a score from 1 to 5 on the Likert scale for each statement [10] (Figs. 1 and 2).

We report the educational program of the courses (Table 1) and the questionnaire for the assessment of the acquired knowledge (Table 2).

**Table 1:** Ultrasound course program

*Introductory part*	
Course introduction	20 min
Teaching ultrasound inside academic curriculum of medical course	20 min
Point of care ultrasound: ultrasound revolution in the clinical approach to the patient	30 min
Ultra sound physics	20 min
Semeiotics, knobology and use of artifacts in ultrasound	30 min
Fundamentals of ultrasound examination (Live lesson)	30 min
Break	20 min
*Ultrasound semeiotics*	
Abdomen overview	30 min
ER ultrasound	20 min
Musculoskeletal system ultrasound in sports	30 min
*Question session*	20 min
Lunch	
*Hands on*	
Hands on training in small groups on healthy subjects	1 h
Training on simulators	1 h

**Table 2:** Questionnaire for the assessment of the acquired knowledge after the ultrasound course

(1) The current transducers are: 1 -Without frequency 2 -With self-regulated frequency in relations to the images on the display 3 -Monofrequency 4 -Multifrequency
(2) Before starting the ultrasound examination on the patient 1 -The orientation of the ultrasound probe is indifferent 2 -Check the correct orientation of the ultrasound probe 3 -The orientation of the probe is always with the landmark towards the right 4 -The orientation of the probe is always with the landmark towards the left
(3) Simple cysts are: 1 -Anechoic 2 -Ipoechoic 3 -Both ipoechoic and anechoic 4 -Iperechoic
(4) What do you mean by PRF? 1 -Pulse Repetition Frequency 2 -Probability Reflection Frequency 3 -Pulse Reflection Frequency 4 -Pulse Recognition Frequency
(5) The acronym EFAST stands for: 1 -Echography fast 2 -Emergency fast 3 -Extended fast 4 -Early fast
(6) The normal diameter of the inferior vena cava: 1 -It should be measured 2 cm from the outlet in right atrium in longitudinal scan 2 -It should be measured 2 cm from the outlet in right atrium in transverse scan 3 -It is indicative of the presence or the absence of ascites 4 -It is not indicative of the patient’s hemodynamic compensation status
(7) B lines are: 1 -Mostly present in case of pnx 2 -Vertical lines visible on the echotorax of a normal 3 -Horizontal artifacts 4 -Abolished in case of acute pulmonary edema
(8) The bedside ultrasound, integrated into the clinic in critical patients of an Emergency Room, carried out by doctors who treat them, has the function of: 1 -Increase confidence in diagnosis 2 -Collect pathophysiological elements 3 -Follow the effect of their own interventions (drugs, procedures) 4 -All the answers above
(9) Following an important osteo-articular sport injury, which investigation should the patient generally undergo after clinical examination? 1 -Radiography 2 -Echography 3 -Computed tomography 4 -Magnetic resonance
(10) How does a mammary cyst appear on ultrasound? 1 -Isoechoic to adipose tissue with clean and regular borders 2 -Isoechoic to mammary glandular corpus with clean and regular borders 3 -Iperechoic with clean and regular borders 4 -Anechoic with clean and regular borders
(11) Which is the most appropriate year of the degree course in Medicine and Surgery for teaching ultrasound? 1 -III 2 -IV 3 -V 4 -VI

## Statistic analysis

The mean of the scores related to the answers of both the evaluation questionnaire of knowledge and the evaluation questionnaire was calculated. In the two cases of Chieti and Rome, in which a comparison between the situations prior to the course and the one immediately following was made, we calculated the differences in the scores achieved and the increase, both absolute and percentage. Moreover, we calculated the median of the scores obtained, the interquartile range and the *p* value by using the Mann–Whitney test, in order to evaluate the statistical validity of the results and to make an adequate comparison between the two courses. The percentage of students who answered some questions with a score of 4 or higher was also calculated.

## Results

A total of 646 students between the fourth and sixth year of the course were included in the study, some of them in the immediate postgraduate period. Of these 87 took part in the ADE in Chieti, 90 in the SIUMB course in Padova, 139 in the SIUMB course held in Montesilvano (PE), 116 in SIUMB course in Naples, 93 in SIUMB course in Rimini and 124 in SIUMB course in Rome. The analysis of the various courses performed with number of participants and percentage of correct answers to the learning questionnaire was showed in Table 3. There was an 81% of correct response to the learning questionnaire by calculating the mean of 5 SIUMB courses performed.

**Table 3:** Analysis of the various courses performed with number of participants and percentage of correct answers to the learning questionnaire

Course	Participants	Correct answers (%)
Padova	90	80,06
Montesilvano	139	85,17
Napoli	116	82,82
Rimini	93	75,17
Roma	124	81,86

## Padova

In May 2017, a SIUMB course was held in Padova. There was a section dedicated to students and the knowledge acquired by students was assessed through a multiple-choice questionnaire comprising 12 questions. The results were positive, and students answered correctly in 80.1% of cases.

## Montesilvano

In May 2018, a SIUMB course, similar to the previous one, was held in Montesilvano (PE). The knowledge acquired by the students was assessed through a multiple-choice questionnaire comprising 11 questions. The results were positive, and the students answered correctly in 85.2% of cases.

## Napoli

In November 2018, a SIUMB course similar to the previous ones was held in Napoli. Students answered correctly in 82.8% of the cases to the questions presented. In that setting, a questionnaire was also administered to assess the satisfaction and motivation of the participants. On a scale from 1 to 5, participants rated on mean 3.3 for the acquisition of knowledge, 2.9 for the acquisition of skills and 3.6 for the acquisition of motivation. In 75% of cases, the students evaluated the topics treated “relevant” or “very relevant”, thus expressing a considerable interest in the subjects covered. The quality of the course was considered overall good, while the value of the course was estimated excellent in 37% of cases, confirming that courses of this type reflect the students’ expectations.

## Rimini

A SIUMB-SISM training course was held in Rimini in April 2019. Students’ knowledge was assessed through a multiple-choice questionnaire developed on the basis of the previous ones. The results were satisfactory, although slightly lower than the other courses, with a percentage of correct answers of 75.8%.

## Chieti

In May 2019, an ADE was held at the “G. d’Annunzio University” in Chieti, consisting of a first theoretical part and a second practical one with a training on voluntary subjects.

Participants were already starting with high levels of motivation and satisfaction, and therefore considering these two parameters there are no significant changes in the results of the questionnaires. The most important differences occur on questions 1 and 3, where there is a percentage increase of 9.9% and 14.7% respectively. By performing a more specific analysis and dividing the participants according to the year of study, the following results emerged regarding questions Q1 and Q3. The percentage increase in the results of the answers for question Q1 is therefore the following:

- 8.6% for fourth year students,
- 9.7% for those in the fifth year,
- 11.8% for those in the sixth year.

Overall, this increase is therefore 9.9% (*p* < 0.001).

The percentage increase for the Q3 response was the following:

- 14.8% for fourth year students,
- 17% for those in the fifth year,
- 10.9% for those in the sixth year.

Overall, this increase is therefore 14.7% (*p* < 0.001). The last three questions, relating to the comparison between the theoretical and practical part and the actual usefulness of the training, also gave positive results, comparable to the previous.

## Rome

The last course analyzed in this study was held in November 2019. A questionnaire for the evaluation of knowledge was delivered, which resulted in a percentage of correct answers of 81.9%. By analyzing the results of the questionnaire evaluating the perception of the course by the students the only two questions for which there was a significant difference between the two forms, A and B, were 1 and 3.

As regards the first one, there was an overall percentage increase of 21.3%. We also analyzed data stratifying according to the year of the course and it emerged that this increase is:

- 21.7% for fourth year students,
- 23.1% for those in the fifth year,
- 19.2% for those of the sixth.

Overall, this increase therefore stood at 21.3% (*p* < 0.001).

The percentage increase for question Q3 was as follows:

- 17.4% for fourth year students,
- 17.4% for those in the fifth year,
- 16.6% for those in the sixth year.

**Fig. 3:**
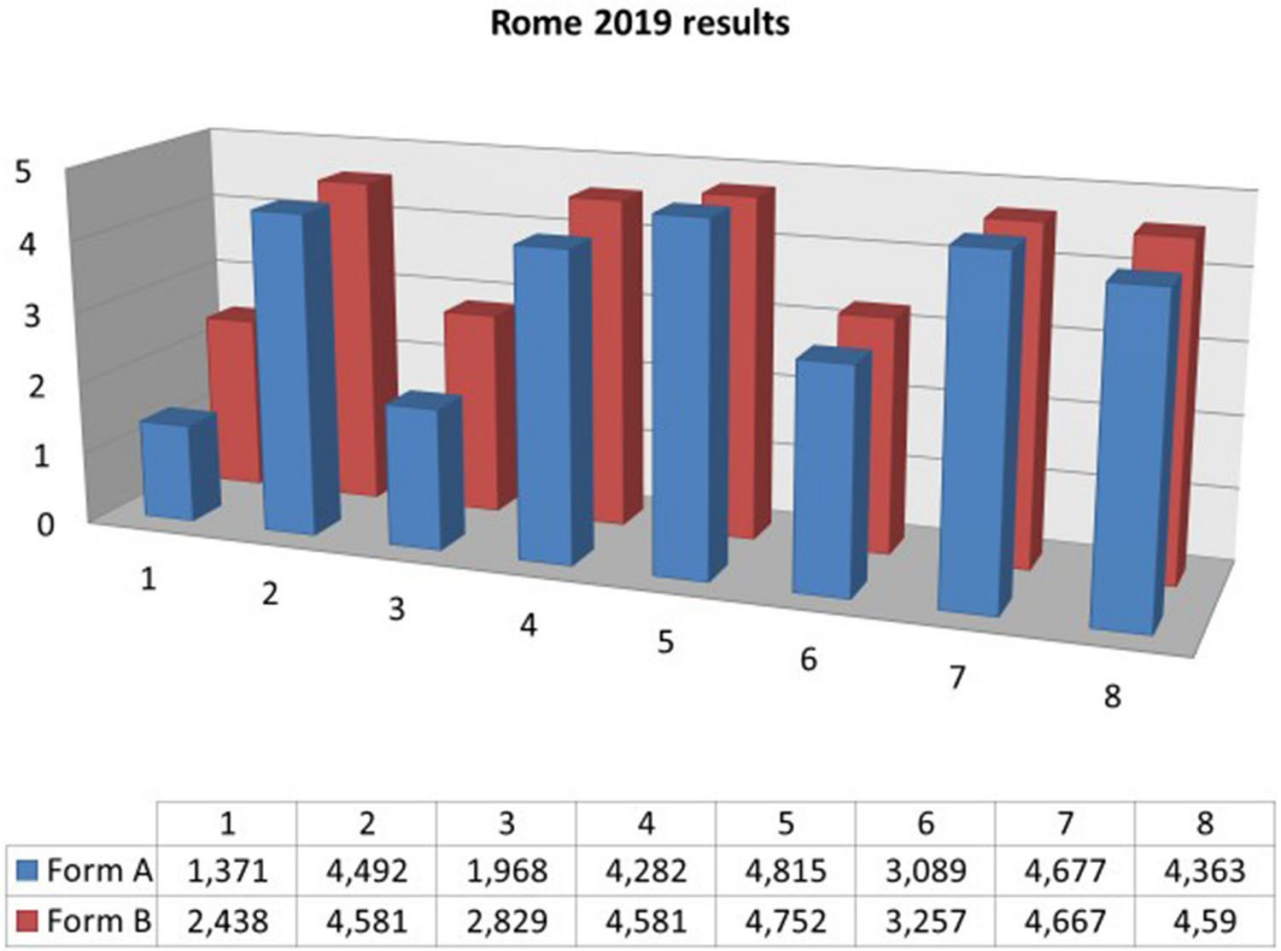
Comparison between the answers to the first eight questions of the questionnaires FORM A and B; data from the SIUMB Course in Rome 2019

There is therefore an overall increase of 17.2% (*p* < 0.001). As regards the other questions, high and scarcely variable results are observed between Form A and B. Regarding Q2, related to motivation, over 90% of the participants answered with a score equal to or greater than 4 in Form A, only slightly increased in Form B. Similar results also occur for Q7, relating to the need for ultrasound courses within the course of degree in Medicine and Surgery. The results of the last three questions are positive, with a mean above 4 (Fig. 3).

## Discussion

Our data show how the students are strongly motivated to continue learning ultrasound already from the beginning of the course, and this result remains unchanged in the questionnaire administered at the end. This could be due to a selection bias, as it is likely that only students already interested in ultrasound would have chosen to participate. On the other hand, that data shows how the interest of students towards this method is high; it is in agreement with the results of Q7, showing how students want ultrasound courses within the of degree in Medicine, even before participating in the training.

However, the most significant data undoubtedly refers to questions Q1 and Q3, relating respectively to the acquisition of skills and knowledge. Since the course in Napoli, in 2018, it was evident how students positively assessed the course in relation to these variables, albeit with a tendency to acquire knowledge rather than skills. However, those data related only to a survey carried out after the course. Therefore, the confirmation was obtained from the courses in Chieti and Rome, which allowed to study the differences between before and after the training. In both cases, there was a significant increase in the score attributed to questions Q1 and Q3. This increase was greater for the Rome course, but a similar result was expected given the different structure and duration of the two events.

Although there was a quantitatively greater increase regarding both answers, the data related to Rome course showed a particularly significant increase for the first question, the one related to skills, while the increase in the values on the third question was only slightly higher than that registered for the Chieti congress (17.2% against 14.7%). These differences are probably related to the different structure of the courses. In both cases, there was a significant theoretical part, which probably impacted the results of Q3 in a similar way. However, by having only one ultrasound machine available, during the ADE in Chieti the practical part was not as effective as it was in Rome, where participants were divided in several small groups; the increase recorded for Q1 was inferior in Chieti course.

The stratification by course year, however, did not lead to univocal results. By considering absolute value, sixth year students begin and end courses with the highest scores, while those attending the fourth year performed lower score referring to Q1 and Q3; however, the increase is variable depending on the case.

Regarding the Rome congress, fourth and fifth year students seem to gradually improve (21.7% against 23.1% for Q1 and 17.4% in both cases for Q3), while maintaining a slight difference in terms of absolute value; the sixth year seems to benefit from the course to a lesser extent. Students from Chieti, on the other hand, showed this trend only for the third question, in which participants attending the fifth year showed the most significant increase. Instead regarding the first question there is an increasing gradient from the fourth to the sixth year.

Those differences may be due both to the different structure of the course and to the different setting of the study in the two universities and to a numerical bias due to the limitation of the sample examined, only partially representative of the general student population.

**Fig. 4:**
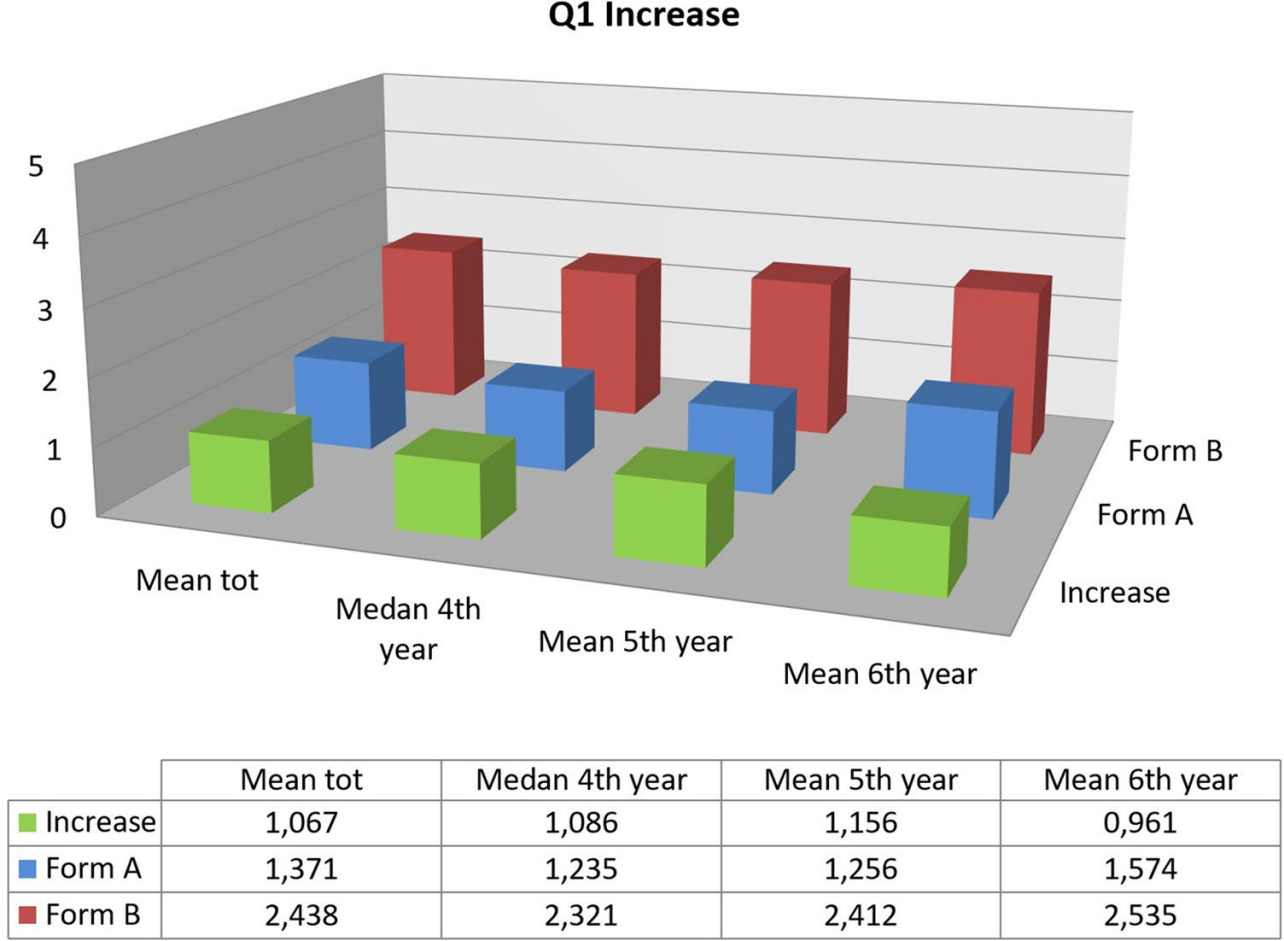
Overall comparison between the answers to the question Q1 “I have already used the ultrasound method acquiring good competences” before and after the ultrasound course with subdivision by year of Medicine degree

**Fig. 5:**
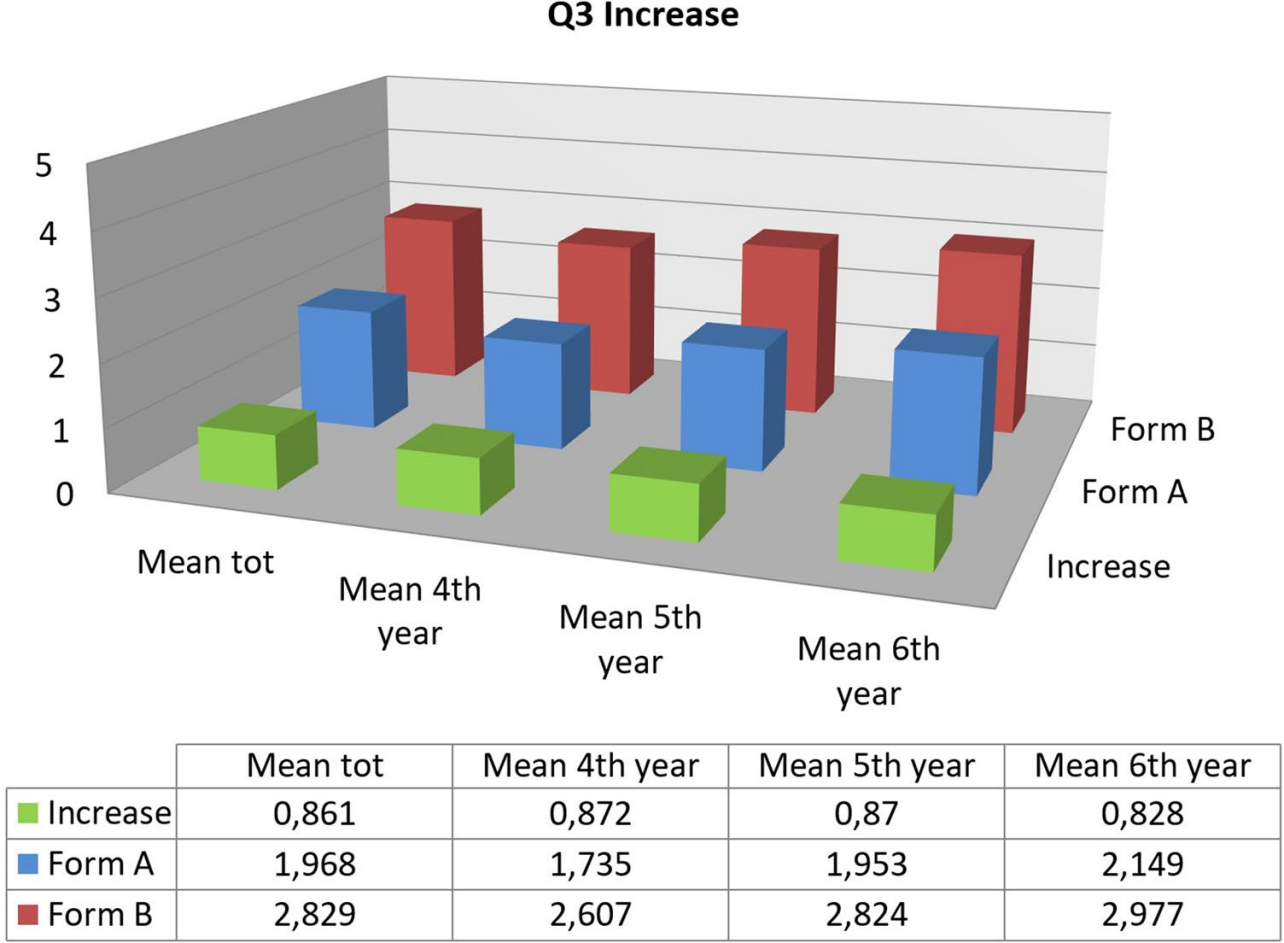
Overall comparison between the answers to the question Q3 “I have technical knowledge for my professionalism regarding ultrasound diagnostics” before and after the ultrasound course with subdivision by year of Medicine degree

As for the last three questions, there were no significant differences between the two courses, confirming that both were able to increase students' knowledge and that the theoretical part resulted useful during the practical one. By analyzing the overall results of the questionnaires evaluating the acquired knowledge administered during the SIUMB courses, positive and comparable results were evident (Figs. 4 and 5).

## Conclusions

Our results support the usefulness of including ultrasound into the curriculum of medical students and on its use as a teaching tool. The non-invasiveness, the possibility of application in numerous fields, the relative simplicity of use and the enthusiasm shown by the students in learning make it an ideal tool. Hands-on part is necessary in the training course on ultrasonography. Students are highly motivated and perceive a significant improvement in both skills and knowledge following the proposed courses.

The use of new technologies in teaching ultrasound and more generally health care also appears effective and appreciated. Their use presents several advantages but with a modification of the traditional approach to education, therefore requiring adequate planning by the universities, in order to apply these resources in the most suitable ways to local needs. Definitive results are still to be assessed and further studies are required, as well as the continuation of this method of training, in order to clarify the most suitable methods and contexts for the application of an instrument that is increasingly important in the context of medical learning.

## Data Availability

The study data are available and can be requested via email from the corresponding author.
